# Efficacy of educational interventions on improving medical emergency readiness of rural healthcare providers: a scoping review

**DOI:** 10.1186/s12913-024-11116-7

**Published:** 2024-07-25

**Authors:** Anju Sreeram, Ram Nair, Muhammad Aziz Rahman

**Affiliations:** 1 Federation University Australia, Mt Helen Campus, Ballarat, Australia; 2 JeevaRaksha Trust, Bangalore, India; 3 Institute of Health and Wellbeing, Federation University Australia, Berwick campus, Berwick, Australia

**Keywords:** Rural healthcare providers, Medical emergencies, Educational interventions, Scoping review

## Abstract

**Background:**

Medical emergencies are the leading cause of high mortality and morbidity rates in rural areas of higher and lower-income countries than in urban areas. Medical emergency readiness is healthcare providers’ knowledge, skills, and confidence to meet patients’ emergency needs. Rural healthcare professionals’ medical emergency readiness is imperative to prevent or reduce casualties due to medical emergencies. Evidence shows that rural healthcare providers’ emergency readiness needs enhancement. Education and training are the effective ways to improve them. However, there has yet to be a scoping review to understand the efficacy of educational intervention regarding rural healthcare providers’ medical emergency readiness.

**Objectives:**

This scoping review aimed to identify and understand the effectiveness of educational interventions in improving rural healthcare providers’ medical emergency readiness globally.

**Methods:**

The Preferred Reporting Items for Systematic Reviews and Meta-Analyses extension for scoping reviews were used to select the papers for this scoping review. This scoping review was conducted using MEDLINE, CINHAL, SCOPUS, PUBMED and OVID databases. The Population, Intervention, Comparison and Outcome [PICO] strategies were used to select the papers from the database. The selected papers were limited to English, peer-reviewed journals and published from 2013 to 2023. A total of 536 studies were retrieved, and ten studies that met the selection criteria were included in the review. Three reviewers appraised the selected papers individually using the Joanna Briggs Institute [JBI] critical appraisal tool. A descriptive method was used to analyse the data.

**Results:**

From the identified 536 papers, the ten papers which met the PICO strategies were selected for the scoping review. Results show that rural healthcare providers’ emergency readiness remains the same globally. All interventions were effective in enhancing rural health care providers’ medical emergency readiness, though the interventions were implemented at various durations of time and in different foci of medical emergencies. Results showed that the low-fidelity simulated manikins were the most cost-effective intervention to train rural healthcare professionals globally.

**Conclusion:**

The review concluded that rural healthcare providers’ medical emergency readiness improved after the interventions. However, the limitations associated with the studies caution readers to read the results sensibly. Moreover, future research should focus on understanding the interventions’ behavioural outcomes, especially among rural healthcare providers in low to middle-income countries.

**Supplementary Information:**

The online version contains supplementary material available at 10.1186/s12913-024-11116-7.

## Background

A medical emergency constitutes a severe injury or any acute illness that requires immediate attention to save a life or to reduce a permanent disability of an individual [1, 2].WHO highlighted that cardiovascular disorders, respiratory disorders and injuries are the major emergency medical causes of death worldwide [2]. A study on global medical emergency disease and burden from 1990 to 2015 shows that 28.3 million deaths globally happened due to emergency medical diseases. Among that emergency medical disease contributed to 25% of deaths in higher-income countries, 35% in upper-middle-income countries, 48% in middle-income countries and 53% in low-income countries [3]. A higher mortality rate related to medical emergencies was observed more in rural areas of low-middle-income countries than in higher-income countries [3, 4].

Worldwide, rural populations are more harmfully affected by medical emergencies than urban populations. It might be related to the geographic density of the rural population. A survey on the global distribution of rural population estimated that 19% of the total population of higher-income countries are living in rural areas of those countries [5]. While the distribution of rural population in low-and-middle-income countries, lower-income countries, lower-middle-income countries, middle-income countries and upper-middle-income countries were 48%, 66%, 57%, and 32%, respectively [5]. Though there is a disparity in the distribution of rural populations between higher-income and middle- to lower-income countries, evidence suggests that rural or remote populations in all countries experience poor availability, accessibility, affordability, and poor-quality health care [4, 6]. Evidence also shows that the occurrence of medical emergency-related morbidity and mortality is higher in rural areas of high-income countries and low to middle-income countries than in urban areas. The identified reasons for the inequitable distribution of healthcare and high mortality rate among the rural populations are the lack of skilled and knowledgeable workforce, equipment and funding [6], lack of effective tools to identify the gaps in medical emergency and provision of care in rural areas health care provision [7] or lack of policies and guidelines regarding emergency care [8–12].

World Health Assembly emphasises the importance of strengthening the emergency health care system to provide timely care for people diagnosed with an acute medical emergency to reduce the ill health or death caused by such emergency conditions. One prerequisite for creating such a system is developing a skilled and knowledgeable workforce who can meet patient needs in an emergency [4, 13, 14]. Educational interventions play an important role in developing a capable rural workforce. Such interventions can enhance rural healthcare providers’ medical emergency readiness [6, 8, 13–16]. Medical emergency readiness is actualising emergency preparedness in an acute emergency [17]. Therefore, rural healthcare providers’ medical emergency readiness constitutes the effective implementation of medical emergency-related knowledge and skills in an acute emergency to save an individual’s life [15].

Rural healthcare providers provide first-line emergency care for emergency patients [18]. However, rural healthcare providers are only partially equipped to provide quality care in low-income and high-income settings. In low to middle-income countries, most of the population lives in rural areas [19]. At the same time, the rural population is increasing in high-income countries with ageing and complexities of medical conditions [20]. However, in both settings, the readiness of healthcare providers for medical emergencies is limited concerning knowledge, skills, and competencies [21, 22]. Many rural healthcare providers have not undergone formal emergency training [22], which will prevent the healthcare providers from providing effective care coupled with emergencies. Therefore, it is essential to train rural healthcare providers to enhance their skills, knowledge and attitudes to provide quality care.

Evidence suggests that rural healthcare professionals’ medical emergency readiness needs an urgent call for attention. For example, one study reported that rural Asian physicians’ cardiac and pulmonary rehabilitation skills and knowledge are limited despite having positive attitudes towards managing medical emergencies [23]. Australian and Chinese studies identified that rural healthcare professionals need more knowledge and skills to manage respiratory medical emergencies [24, 25]. An African study highlighted that the high antenatal and post-natal maternal mortality rate was associated with healthcare providers’ poor knowledge and skill to manage such emergencies [26]. A US study identified that an inadequately prepared workforce could not provide support during emergencies and emphasised the importance of education and training [27]. An Australian study showed that rural health nurses need a greater understanding of trauma-informed principles and practices [28]. Another study shows that rural healthcare professionals need more knowledge and skills to manage prehospital psychiatric emergencies [29]. Rural healthcare professionals had limited theoretical knowledge and practical skills to manage life-threatening emergencies in rural areas compared to urban healthcare professionals [30]. The above evidence indicates the importance of strengthening the rural health carers’ medical emergency readiness.

Rural populations worldwide are affected by high mortality and morbidity with medical emergencies. One of the reasons for this incidence is the need for more medical emergency readiness among rural healthcare professionals. Evidence shows educational interventions will effectively improve rural healthcare professionals’ medical emergency readiness. Despite those findings, research on rural healthcare providers’ medical emergency readiness is limited. Therefore, this review aims to explore rural healthcare providers’ medical emergency readiness and identify the effect of educational interventions to enhance rural healthcare professionals’ knowledge, skills and competency to provide timely care in acute medical emergencies in rural health settings. Systematic reviews on prehospital emergency readiness of healthcare professionals show that they were not effectively prepared to provide efficient care during medical emergencies and emphasise the importance of training needs for rural healthcare providers [14, 18]. So far, a review has yet to be reported about the effectiveness of educational training to enhance rural healthcare providers’ medical emergency readiness. Therefore, a scoping review is conducted to understand the effectiveness of educational intervention in improving the medical emergency readiness of rural healthcare providers.

## Methods

The scoping review aimed to understand the efficacy of educational interventions in improving rural healthcare providers’ medical emergency readiness. The methodology used for this scoping review is Preferred Reporting Items for Systematic Reviews and Meta-Analyses extension for scoping reviews [PRISMA] [19]. The scoping review helps to map and summarise the available literature about the selected study, explore gaps in the selected research areas and direct to future research. Moreover, scoping reviews are helpful when the literature is complex and heterogeneous [19–21]. This review followed Joanna Briggs Institute’s [JBI] scoping review methodology, Preferred Reporting Items for Systematic Reviews and Meta-Analyses extension for scoping reviews [PRISMA] [19]. The JBI scoping review provides contemporary guidance to researchers regarding scoping review based on the topic, analysis, presentation, implication for practices and reduction of bias [22]. Simultaneously, it mirrors with PISMA-ScR [19, 22]. This scoping review aims to identify and analyse the peer-reviewed scientific literature focusing on rural healthcare providers’ medical emergency readiness and the effectiveness of educational interventions to enhance medical emergency readiness.

### Search strategy

A qualified librarian assisted in identifying the selected studies from the database. The database was MEDLINE, CINHAL, SCOPUS, PUBMED and OVID. We searched the database from January to September 2023. The selected studies were limited to English and published between 2013 and 2023 in peer-reviewed journals with full-text articles. A comprehensive search using the database will reduce the selection bias and to understand the current drift in rural healthcare providers’ medical emergency readiness. Evidence shows that a sufficient number of databases will minimise the selection bias and improve the validity and generalisability of the review results [31]. Therefore, the search strategies used in this review may reduce the selection bias and to understand the current drift in rural healthcare providers’ medical emergency readiness. The selected studies were identified using Population, Intervention, Comparison, and Outcome [PICO] strategies. Search terms were rural OR remote “health care providers “OR “health care clinicians” OR “doctors” OR “nurses” AND “educational intervention” OR “training” OR “basic life support training” OR “Evidence-based practices” AND medical emergency readiness OR competence OR skill OR knowledge or confidence AND effect of intervention OR training OR education OR evidence-based practice OR basic life support*. See Appendix 1 for PICO strategies.

### Eligibility criteria and study selection

This review included all identified original articles focused on medical emergency readiness-related interventions based on PICO strategies. Therefore, the paper included Randomised Controlled Trials [RCT] and quasi-experimental designs. At the same time, we excluded abstracts, qualitative studies, protocols, pilot studies, grey literature, narrative, and systematic reviews, which did not follow PICO strategies in this review. All identified studies were uploaded to Endnote, and duplicates were removed. Finally, ten studies were included in the final review. The selected studies were appraised using the Joanna Briggs Institute [JBI] critical appraisal tool for randomised controlled and quasi-experimental trials to rule out the risk of bias [32]. Those studies were appraised by three individual appraisers using the JBI tool to assess methodology, study design, interventions, analysis, and bias in the selected documents. See appendices 2&3. The selected ten papers were sent to the research team. Three team members independently appraised each of them using JBI tool. Individual critical appraisal helps to understand the quality of the selected paper, design, research process, analysis and bias in the selected studies. All team members appraised eight selected papers using the quasi-experimental critical appraisal tool [9, 33, 23, 32–35], and the other two papers were reviewed using the randomised controlled critical appraisal tool [36, 37]. Subsequently, the team came together and discussed the reasons for including those papers in the review. After resolving the discrepancies, ten studies were unanimously selected for review.

### Data extraction and synthesis

We included randomised and non-randomised experimental studies regarding rural healthcare providers’ medical emergency readiness. Our search considered all interventions focusing on improving adult, paediatric, obstetric, and gynaecological as well as psychiatric emergency readiness and outcomes of those interventions of the readiness of rural health care providers.

The review used a descriptive approach to synthesise the data from the selected studies. Narration and tabulation are used for data extraction and analysis in a descriptive approach [38]. The narrative synthesis and tabulation help organise the selected studies’ findings and understand the interventions’ effect [39, 40]. The analysis of selected studies showed that population, emergency context, outcome measures, interventions, and analysis methods varied in nature. Therefore, this review used a descriptive approach instead of a meta-analysis [41].

## Results

**Fig. 1:**
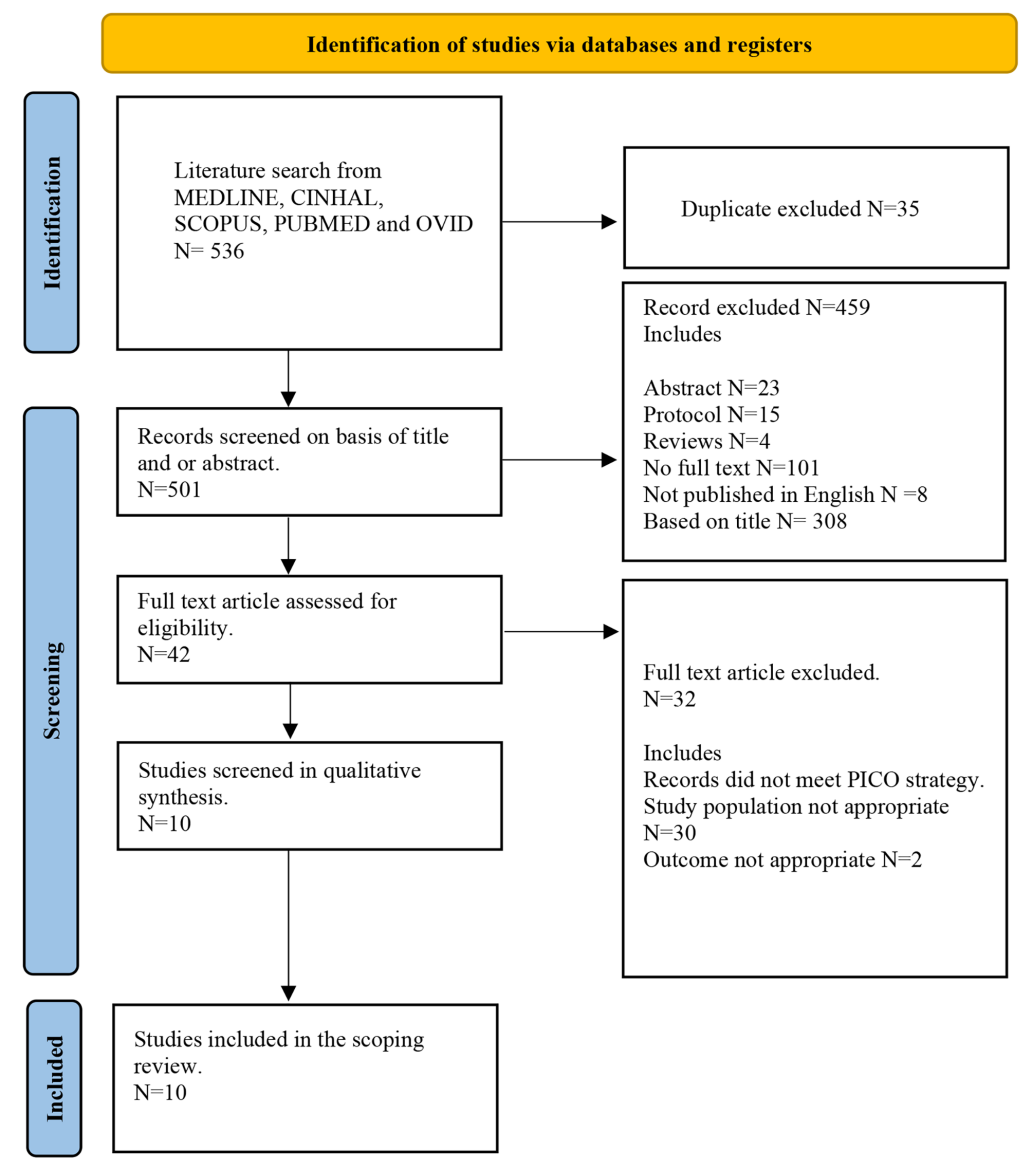
Prisma flow chart

The search yielded 536 studies. After removing the duplicate, 501 studies were selected for screening. Those 501 studies were screened based on the PICO strategies and 42 articles were reviewed completely. Finally, ten studies were included in the final review. Figure 1 PRISMA flow chart shows the inclusion and exclusion criteria of the selected emergency studies.

### Characteristics of studies

The selected eight studies used a quasi-experimental design [33, 23, 32–35, 42, 43]. Two studies used RCT design [36, 37]. Most published studies were from higher-income countries [9, 32, 35, 37, 42, 43]. One study was from an upper-income country, and the rest were from lower-income countries [23, 33, 34, 36]. Among the ten selected studies, four were entirely focused on adult medical emergency readiness [23, 32–34], and three were about the paediatric medical emergency readiness of rural healthcare providers [9, 37, 43]. The remaining studies dealt with psychiatric and obstetric emergency readiness [35, 36, 42].

Five studies conducted follow-up studies to understand the sustainability of educational intervention [32, 35, 36, 42, 43]. Whilst remaining engrossed in the immediate effect of the interventions [9, 23, 33, 34, 37], four studies reported improvement in teamwork with the training [9, 32, 33, 42]. One study pointed out that years of experience in managing emergencies could enhance knowledge levels [33]. Table 1 summarises the review results.

**Table 1:** Summary of the results

Author Year Country	Emergency care area	Participants	Research design	Phases			Interventions	Results	Outcomes
				Pre-test (*n*)	post-test (*n*)	Follow up. (*n*)			
Christiansen et al., (2023), Ethiopia [36]	Obstetric emergency	Rural healthcare providers	RCT	176	176	130	1 h with safe delivery app [SDA], simulation and scenario-based training.	Skill: 0–6 months: intervention group M = 29.9; Control Group M = 1.8, D = 27.8, 6–12 months Intervention group M = 13.3, Control M = 3.1, D = 27.8 0–12 months: Intervention group M = 42.9, Control M = 4.9, D = 38. Knowledge: 0–6: Intervention M = 8.5, Control M = 1.9, D = 6.6 6–12 months: Intervention M = 3.0, Control M = 1.1, D = 1.9. 0–12: intervention M = 11.5, Control M = 3.0, D = 8.5.	The experimental group acquired more skill and knowledge than the control group, and the experimental group could sustain the knowledge and skill
Cullinane et al., (2022) Australia [42]	Maternal and Neonatal Emergency	Rural healthcare providers	Quasi-experimental design	294	120	90	One-day onsite Maternity and Newborn Emergencies [MANE] program	Knowledge of teamwork principles and how to communication: pre-MANE = 50%; post MANE = 60%; 6 months post MANE = 55%;12 months post MANE = 68%. Knowledge of clinical governance and risk management: pre-MANE = 25%; post MANE = 58%; 6 months post MANE = 59%;12 months post MANE = 58%. Knowledge of eclampsia management: pre-MANE = 25%; post MANE = 59%; 6 months post MANE = 41%;12 months post MANE = 61%. Knowing how to escalate and access help: pre-MANE = 49%; post MANE = 50%; 6 months post MANE = 50%;12 months post MANE = 50%. Knowledge of situational awareness and access to resources: pre-MANE = 38%; post MANE = 60%; 6 months post MANE = 58%;12 months post MANE = 50%. Knowledge of post-partum haemorrhage management: pre-MANE = 32%; post MANE = 58%; 6 months post MANE = 55%;12 months post MANE = 62%. Knowing how to take leadership and when to delegate: pre-MANE = 35%; post-MANE = 59%; 6 months post-MANE = 55%;12 months post-MANE = 54%. Knowledge and understanding of performing newborn resuscitation: pre-MANE = 30%; post MANE = 58%; 6 months post MANE = 55%;12 months post MANE = 45%. Confidence in managing eclampsia: pre-MANE = 20%; post MANE = 62%; 6 months post MANE = 64%;12 months post MANE = 82% Confidence post-partum haemorrhage: pre-MANE = 45%; post MANE = 82%; 6 months post MANE = 99%;12 months post MANE = 95%) Confidence in newborn resuscitation: pre-MANE = 41%; post MANE = 82%; 6 months post MANE = 83%;12 months post MANE = 85%. Stress level in managing Eclampsia after MANE program: pre-MANE = 59%; post MANE = 70%; 6 months post MANE = 63%;12 months post MANE = 59%. The stress level in managing post-partum haemorrhage after MANE program: MANE = 59%; post MANE = 63%; 6 months post-MANE = 50%;12 months post MANE = 50%. The stress level in managing Newborn resuscitation after the MANE program: pre-MANE = 58%; post-MANE = 59%; 6 months post-MANE = 55%;12 months post MANE = 60%.	Knowledge, Confidence, teamwork and leadership quality improved after the intervention. There was no change in related stress levels after the intervention.
Ferguson et al., (2018) Australia [35]	Psychiatric emergencies	Rural healthcare providers	Quasi-experimental design	236	176	no	One-day suicide prevention education session with lived experience.	Attitude scale items scores Item 1: Z=-3.03, *p* = .002Item 2: Z= -3.21, *p* = .001 Item 4: z=-3.57. p=‹0.0001 Item 5: z=-11.32, p=‹0.0001 Item6: z=-4.38, p=‹0.0001 Item 8: z=-2.73, *p* = .006 Item 10: z=-4.55, p = p=‹0.0001 Item 11 = z=-6.64, p=‹0.0001 Item 12: z=-3.0, *p* = .001 Item 13: z=-3.23, *p* = .001 Item 14: z=-5.26, p=‹0.0001 Confidence scale scoring Item 1: z = 12.45, p=‹0.0001 Item 2: z = 12.45, p=‹0.0001 Item 3: z = 11.32, p=‹0.0001 Item 4: z = 19.23, p=‹0.0001	Rural Healthcare providers’ attitudes and confidence level to manage suicide prevention improved.
Gutenstein et al., (2019) NZ [32]	Adult trauma emergency	Rural healthcare providers	Quasi experimental design	60	60	40	Three Days Rural Interprofessional Simulation course (RiSC) training	Effect on learning experience: *N* = 40; 39% Effect on valuable rural networking *N* = 40;35% Effect on realistic interprofessional relationships = 40;33% Effectiveness of simulation = 40;36% Effect of debriefing: *N* = 40;34% Effect on Experience; *N* = 40,28% Effect on Safe learning environment: *N* = 40;37% Future improvement in clinical practice: *N* = 40;36% The expectation of learned principle in future practice: *N* = 40;29%	Improved skill, teamwork, and Confidence after the training
Kivlehan et al., (2021) Uganda and Tanzania [34]	Medical emergency	Rural healthcare providers	Quasi experimental design	59	59	no	Five days intensive training, World Health Organization-International Committee of the Red Cross Basic Emergency Care course. (WHO-ICRC-BECC).	Emergency Knowledge: *N* = 57, pre-M = 14, post M = 19, V = 1372, *P* ≤ .001. Emergency confidence: *N* = 48, Pre-M = 3.07, Post M = 3.81t = 14.19, p = *P* ≤ .001.	Knowledge and Confidence improved after the intervention.
Menon et al., (2023) India [23]	Medical emergency	Rural Physicians	Quasi experimental design	622	622	no	3-hr researcher led training	Cardiac resuscitations Knowledge score = 622 Pre-M = 1.5, Post M = 10.5, *P* = .0001. Cardiac resuscitation skills: *N* = 622, Pre- M = 0.1, post-m = 2.8. *p* = .0001	Physicians’ cardiac resuscitation knowledge and skills have improved after the intervention.
Monachino et al., (2019) The US [9]	Paediatric medical emergency	Rural healthcare providers	Quasi experimental design	211	191	no	2-hour instructor-led program for 14 months in 30 PHC.	Doctors’ confidence level: pre-*N* = 47, post *N* = 47; Pre-M = 3.83; Post M = 4.32, *p* = .003. Doctors Competence Pre-M = 3.76, post M = 4.3; *p* = .000. Nurses’ Confidence pre *n* = 71, post *N* = 67 Pre M = 3.53.post M = 4.03. *p* = .000 Nurses’ Competence pre-M = 3.69, post M = 4.15, *p* = .000. Medical assistants’ Confidence: pre-*N* = 16 post-*N* = 13 Pre-M = 4.15, Post M = 4.5, *p* = .094 Medical assistants’ competence pre-M = 4.15, post M = 4.56, *p* = .107 Patient service representatives’ Confidence: pre-*N* = 65, post-*N* = 51 pre-M = 3.8, post M = 4.24 p=,002. Patient service representatives’ competence pre-M = 3.75, post M = 4.25 *p* = .000 Others confidence: pre-*N* = 12, post *N* = 10 Pre-M = 3.67, post M = 4.4 *p* = .003. Others Competence: pre-*N* = 12, post *N* = 10 pre-M = 3.58, post M = 4.5 *P* = .002	Confidence and competency level of paediatric emergency management of rural health care providers have been improved.
Naidoo (2017) South Africa [33]	Adult medical emergencies	Rural healthcare providers	Quasi experimental design	321	211	no	Two days of collaborative educational interventions led by two doctors and two nurses	Resuscitation Pre-M = 2.17, Post M = 4.27 Trauma: Pre-M = 2.79, Post M = 4.02 Cardiac emergencies: Pre-M = 2.60, Post M = 3.89 Medical emergencies: Pre-M = 2.85, Post M = 3.96 Sexual assault: Pre-M = 2.53, Post M = 3.75 Unconscious patient: Pre-M = 2.89, Post M = 4.05 Toxicology: Pre-M = 2.2.56, Post M = 3.86 Paediatrics: Pre-M = 2.73, Post M = 3.86 HIV emergencies: Pre-M = 2.64, Post M = 3.86 Radiology: Pre-M = 2.42, Post M = 3.85 Between participants Nurses: Pre-M = 4.3, Post M = 6.63 Doctors: Pre-M = 3.72, Post M = 5.57.	Improvement in skill and knowledge after the training.
Shenoi et al., (2013). The US [43]	Paediatric emergencies	Rural healthcare providers	Quasi experimental design	23	23	14	1 h training with researchers.	Physicians’ knowledge score: pre-M = 11.88 post M = 13.71, t = 1.83, P=‹0.002 d = 0.98 Nurses’ knowledge score: pre M = 10.85.post M = 11.44.t = 0.59, *p* = .13 d = 0.24. Physicians’ comfort in managing emergencies pre-M = 6.88, post-M = 8.17, md = 1.3, p=‹0.002, d = 0.84. Nurses’ comfort in managing emergencies pre-M = 6.04, post-M = 7.44, md = 1.4, p=‹0.004. 80% sustainability of acquired knowledge after three years.	Knowledge and competency levels improved immediately after the intervention, with 80% sustainable results three years later.
Stellflug et al., (2017). The US [37]	Paediatric emergencies	Rural healthcare providers	RCT	No pre-test	94	69	2-day Paediatric Advanced Life Support [PALS] course with high-fidelity and low-fidelity manikins.	Knowledge level between experimental and control groups immediately after the intervention: Post -M (immediate) experimental group = 29.74. Post-M (immediate) control group = 29.63, Knowledge six-month post-training, between experimental and control group, experimental M = 27.71, control M = 26. 23. Emergency management skill between experiment and control group immediately after the intervention: Post M (immediate) experimental group = 8.5. Post M (immediate) control group = 8.6, Emergency management skill between experiment and control groups six months later: experimental M = 8.3. control M = 7.3 Core case scenario time to task recognition between both groups: experimental group M = 99.9; control group M = 112.6. Core case scenario time to intervention between both groups: experimental group M = 140.7, control group M = 158.6. Core case scenario Time to reassessment between both groups: experimental group M = 154; control group M:186.	The knowledge and skill levels of the participants trained with high-fidelity and low-fidelity manikins were the same. Improvement in time management was more apparent with high-fidelity manikins.

### Medical emergency readiness of rural health care providers

Emergency readiness of the healthcare providers was identified based on their knowledge, attitudes, skills, and confidence. Nine studies explored rural healthcare providers’ knowledge about managing various medical emergency readiness [9, 23, 32–34, 36, 37, 42, 43]. One study addressed attitudes towards managing psychiatric emergencies [35]. Six studies tried to understand rural healthcare providers’ skills in managing medical emergencies [9, 33, 34, 36, 38, 39]. Six studies explored rural healthcare providers’ confidence levels to facilitate emergency management [9, 31, 32, 35, 37, 39]. All selected studies’ pre-test results identified room for knowledge, skills, attitudes and confidence improvement among healthcare professionals globally.

### The effect of educational interventions regarding medical emergency readiness of rural healthcare providers

In this review, four studies addressed healthcare professionals’ adult medical emergency readiness. One study used the Rural Interprofessional Simulation Course [RiSC] training to enhance knowledge and skills in managing emergency trauma. They also noted that such training effectively improved interprofessional relationships, provided safe learning experiences, and reduced the gap between emergency readiness of rural and urban healthcare providers. RiCS training positively impacted rural healthcare providers’ confidence and competency to manage trauma care in rural healthcare settings [38]. The second study explored the World Health Organization-International Committee of the Red Cross Basic Emergency Care Course [WHO-ICRC-BECC] training and found it effective in managing medical emergencies in rural settings using a train-the-trainer model. The training enhanced rural healthcare providers’ knowledge and confidence in emergency management. While participants informed that it is essential to have such training on emergency obstetric management, training the local trainers is the cost-effective way to reduce expenses in low- to middle-income countries. The result also shows that all rural healthcare staff, including security, cleaners, and administrative office staff, should undergo such training [39]. The third study implemented an interprofessional simulation and lecture-based teaching to assess knowledge and skills regarding medical emergencies. The results showed there was an improvement in knowledge and skill after the training regarding trauma management, Cardiac emergencies, medical emergencies, sexual assault, unconscious patients, toxicology, paediatrics, HIV emergencies and radiology. Nurses’ pre-and post-knowledge level of emergency management was higher due to many years of exposure to emergencies than doctors. The result also emphasised the importance of having regular emergency training in rural settings to sustain the knowledge and skill. Similarly, the study shows the importance of integrating subcutaneous pacing and dermatological emergencies in the training and education of rural healthcare providers. They also reported that the training improved teamwork and interprofessional relationships [40]. The last study focused on the effectiveness of Cardiac Arrest Resuscitation Short [CARS] courses for physicians. The intervention was effective in enhancing Asian rural physicians’ knowledge and skills in cardiac arrest management. The result also showed that cardiac arrest resuscitation short courses were cost-effective. Such interventions will be effective in low-resource settings [33].

Three studies focused on paediatric emergency management [9, 41, 42]. The training programs used in those studies were instructor-led paediatric emergency management program [9], paediatrics office medical emergency preparedness program [41] and Paediatric Advanced Life Support [PALS] Courses with low-fidelity manikins (control group) and high-fidelity manikins (experimental group) [42]. Those interventions effectively enhanced paediatric healthcare providers’ knowledge, skills, competence, and teamwork. However, two studies reported that the training program, which was developed based on the scope of practice of specific healthcare professionals, yielded more positive outcomes than generally trained healthcare providers [9, 41]. The study compared the effect of high-fidelity and low-fidelity manikins in managing paediatric emergencies [42]. The results showed no difference in acquired knowledge and skills between the control and experimental groups immediately after the intervention. However, the study identified that PAL with high-fidelity manikins was more effective than PAL with low-fidelity manikins in sustaining rural healthcare providers’ knowledge and skills in paediatric emergency management and core case scenario time to task recognition was significantly improved among the experimental group [42]. Two selected studies were focused on the effect of interventions in maternal emergency management [35, 36]. One study focused on rural healthcare providers’ knowledge and skills in maternal emergency management using the Safe Delivery App [SDA]. The result showed that the intervention group acquired more skill and knowledge than the control group during each interval [35]. The second study was focused on implementing and evaluating the Maternal and Neonatal Emergency [MANE] program. The results showed that there was an improvement in various components of knowledge, such as knowledge of teamwork principles and how to communicate effectively, knowledge of clinical governance and risk management, knowledge of eclampsia management, knowing how to escalate and access help, knowledge of situational awareness and access to resources, knowledge of post-partum haemorrhage management, knowing how to take leadership and when to delegate and knowledge and understanding of performing newborn resuscitation. The results also showed that the MANE program was effective in enhancing confidence in managing eclampsia, post-partum haemorrhage and newborn resuscitation. However, the results showed no change in managing the stress level of those conditions after the MANE program. The result also showed positive participant behavioural outcomes [36].

One study focused on psychiatric emergencies and explored the effectiveness of education involving lived experience to manage such emergencies [37]. The result shows that attitudes and confidence of rural health care providers in emergency psychiatric management had been improved immediately after the intervention. However, attitudes and confidence levels remained the same four months later. They highlighted that improved attitudes and confidence may be related to consumer involvement [37].

Few studies focused on the sustainability of acquired medical emergency knowledge, skill, and confidence among rural healthcare providers, and the observational period varied from three months to three years. All follow-up studies results show that the associated intervention based on the emergency was effective in maintaining the sustainability of medical emergency readiness among rural healthcare providers [35–38, 41].

Another interesting aspect identified through this review was the duration of the training. All interventions were implemented in varying periods, from one hour to five intensive days. Three studies conducted one-hour training [35, 36, 41]. Two studies reported two to three hours of training [9, 33]. While other selected studies conducted training for days [37–40, 42]. The results show that despite the variation in time and foci, all interventions effectively improved healthcare providers’ medical emergency readiness.

Another distinct element identified in the educational interventions was the effective use of simulation techniques to enhance healthcare providers’ medical emergency readiness. In which six studies incorporated low-fidelity manikins [33, 36, 38–41] One used a high-fidelity manikin [9]. One study compared the effect of high- and low-fidelity manikins [42]. Maternal emergency training uses a Safe Delivery App [SDA] and low-fidelity manikins to manage maternal emergencies [35]. Mental health emergency training incorporated lived experience [37].

Regarding the application of manikins, one study found that there was no difference in the acquired emergency knowledge and skills by using high and low-fidelity simulation training immediately after the intervention. However, the sustainability of acquired knowledge and skill was higher among those who used high-fidelity manikins than the low-fidelity manikins [42]. Despite variations in the interventions in the selected studies, the results show that all distinct elements identified in the interventions effectively enhanced rural healthcare providers’ medical emergency readiness. All identified studies used statistical methods to analyse the effectiveness of the interventions.

Overall, all interventions were effective in improving rural healthcare providers’ medical emergency readiness. Such interventions not only improved the readiness but also enhanced teamwork and collaborative practices. The worldwide utility of low-fidelity manikins was commendable. However, the interventions designed based on specific healthcare professionals’ scope of practice will be more beneficial.

### Medical emergency readiness of rural health care providers in higher-income and lower to middle-income countries are providers

The identified studies were from different parts of the world. Three studies were from African backgrounds. In which an Ethiopian study explored rural healthcare providers’ obstetric emergency readiness [35]. A South African study focused on the medical emergency readiness of healthcare providers [40]. Another study tried to understand the medical emergency readiness of rural healthcare providers from Uganda and Tanzania [39]. Indian study explored Asian Physicians’ knowledge and skill in cardiac arrest [33]. Two studies from Australia focused on rural healthcare providers’ psychiatric emergency readiness and maternal and neonatal emergency management readiness [36, 37]. The remaining studies were from American backgrounds and explored rural healthcare providers’ paediatric emergency preparedness [9, 41, 42].

Comparing the pre-test results of rural healthcare providers’ medical emergency readiness has shown that there was room for improvement in the medical emergency readiness among healthcare providers from higher and lower-income countries. Nevertheless, all interventions were effective in filling the identified gap in readiness. Therefore, the result concluded that rural healthcare providers’ medical emergency readiness was the same globally.

### Barriers identified for medical emergency readiness and the effect of educational interventions among rural healthcare providers of higher-income and lower to middle-income countries

The review results also illuminated the barriers that can erode rural healthcare providers’ medical emergency readiness. At the same time, there were variations in the barriers identified by the higher and low-middle-income countries’ rural healthcare providers’ medical emergency readiness and the effectiveness of the interventions. The studies from the higher income countries reported that inadequate staffing in rural areas, availability of equipment, attitudes towards medical emergency training and the rare occurrence of medical emergencies in rural areas limited rural healthcare medical emergency readiness [9, 32, 37, 43]. Moreover, studies from higher-income countries reported that such training was not mandatory for the staff to participate. Therefore, healthcare providers never show interest in participating in the training [9, 32]. Meanwhile, low-middle income countries reported a lack of qualified staff, lack of equipment and transport facilities, lack of trainers to train the staff, lack of training and financial burden. These barriers prevent medical emergency readiness of low to middle-income countries’ rural healthcare providers [23, 33, 36].

## Discussion

This scoping review shed light on rural healthcare providers’ medical emergency readiness. The review results showed that rural healthcare providers need more knowledge, skill, and confidence to meet medical emergencies in rural healthcare settings. There was a similarity in the identified gap globally. A previous study focusing on the training needs of Australian rural physicians to manage medical emergencies showed that their medical emergency readiness needed to be improved and emphasised the importance of training needs [30]. Another study conducted in the US also showed that emergency clinicians have knowledge and skill gaps to manage medical emergencies [26]. A Chinese study reported that rural healthcare providers need more knowledge, skill and competency to meet medical emergencies [24]. Another African study reported that the availability of knowledgeable and skilled healthcare professionals is a requisite to manage medical emergencies, and preparing such a workforce for the future in Africa is essential [43].

The review’s focus was understanding the intervention’s effect on the medical emergency readiness of rural healthcare providers globally. The review results showed that all interventions were effective in enhancing rural healthcare providers’ medical emergency readiness [9, 33, 35–42]. These studies explored various emergency knowledge, skills and confidence levels of rural healthcare providers. However, nine out of ten studies’ distinct feature was the application of simulation techniques that might be the reason for improving rural healthcare providers’ emergency readiness. Simulation-based education integrates simulation scenarios, reflections and implementation of learned behaviour. Such educational intervention enables the learners to acquire knowledge, skills, and competence levels more effectively than classroom teaching. Evidence suggested that the simulation-based competency-focused paradigm positively impacted healthcare providers’ knowledge and skills in consumer care [44]. Simulation-based training helps healthcare providers to provide the best patient care [45, 46]. This review also pointed out that training and years of experience in managing medical emergencies are other factors in improving skills and knowledge among healthcare providers [40]. A study on practising nurses’ professional competency aligns with current findings [47]. Moreover, training helped to improve teamwork among healthcare providers [36, 38, 40]. A previous study on interprofessional teamwork in comprehensive primary healthcare services supports the current findings [48].

Another area identified in this review was the usefulness of low-fidelity simulation techniques in rural areas. Seven studies used low-fidelity simulation training and were effective [33, 35, 36, 38–41]. The reasons for implementing low-fidelity simulation in rural healthcare settings may be related to effective budgeting. A previous study on factors affecting the implementation of simulation-based training aligns with the current findings [49]. The review shows that psychiatric emergency readiness of rural health care providers improved with lived experience involvement. A previous study also pointed out that lived experience involvement in the education and training of health professionals is an effective way to enhance mental health knowledge skills and attitudes towards the care of people with mental illness [50].

The period of implementation of educational training was different in each selected study. In this review, training implementation varied from one-hour to five days intensive program [39, 41]. The difference in the duration of training may be related to the number of emergency components they have covered during the training. Nevertheless, the differences in training duration effectively enhanced medical emergency readiness. The reason could be that the programs are designed clearly and concisely to address medical emergencies with simulation. A previous study suggested that disease-specific and evidence-based training will effectively improve healthcare providers’ knowledge and attitudes to care for patients [51]. However, one study in this review identified that paediatric emergency training was more effective for doctors than nurses because the training modules were precisely designed for doctors [41]. Therefore, creating an educational intervention based on the needs of each healthcare provider’s scope of practice would be beneficial. A systematic review of effective training methods to improve patient care aligns with current findings [44].

This review provides information about the medical emergency readiness of rural healthcare providers in higher- and lower-middle-income countries. This review identified ten studies, among which six studies were from higher-income countries [9, 36, 38, 41, 42]; one was from an upper-middle-income country [40], and three were from lower-middle-income countries [33, 35, 39]. The results show that rural healthcare providers’ medical emergency readiness needed to be improved in higher- and lower- to middle-income countries. Another study also pointed out the existing discrepancy in knowledge, skills, and competence levels of rural and urban healthcare providers worldwide [52]. In this review, one study pointed out that educational interventions effectively reduce the gap between the readiness of rural and urban healthcare professionals [38]. Evidence also identified that trauma-related morbidity and mortality rates are higher in rural areas of both higher-income and lower-income countries [4]. Therefore, it is imperative to have knowledgeable and skilled healthcare workers to reduce such occurrences in rural areas, especially in middle- and lower-income countries. Ninety per cent of the people live in rural areas of developing countries. India and China contribute 45% of that population [4]. Evidence shows that the emergency-related mortality rate is high in the Indian subcontinent, and India is accountable for 22% of trauma-related deaths [53, 54], and this may be related to a lack of resources and skilled professionals to meet emergency care [18, 53]. Therefore, it is crucial to identify rural healthcare providers’ medical emergency readiness from these provinces and the development and implementation of intervention-related medical emergency readiness. Future research should focus on these areas.

Finally, this review explored the barriers associated with rural healthcare providers’ medical emergency readiness. Both higher and lower-income countries reported that severe shortage of rural health care providers, lack of qualified staff and trainers, cost associated with training, and limitation of other resources are the main barriers related to rural health care development [9, 35, 38, 39, 42]. Global evidence shows that the inequities in rural health protection are due to staff shortages, unqualified staff and unequal budgeting between rural and urban health care [4]. Two studies reported that the non-occurrence of such emergencies, staff attitudes, and the gap in policies and their implementation related to rural healthcare were the barriers to implementing training for rural healthcare providers [9, 41]. Previous studies on hurdles associated with providing effective emergency medical care align with the current findings. They reported that lack of skilled and knowledgeable staff in rural areas, unavailability of equipment, poor transport facilities, limited funding for rural healthcare and overlooking rural healthcare systems were the main reasons for rural healthcare providers’ medical emergency readiness [6, 8–12].

### Strengths and limitations

This is the first scoping review of rural healthcare professionals’ medical emergency readiness and the effect of education. This review methodology enabled us to explore the emergency medical readiness of rural healthcare professionals and the impact of various interventional programs to enhance rural healthcare professionals’ knowledge, skills, and confidence globally. This review identified that rural healthcare providers’ global medical emergency readiness needed improvement. The identified educational interventions to strengthen rural healthcare providers’ medical emergency readiness were promising.

A competent healthcare team in rural health services can reduce those population’s mortality and morbidity levels. At the same time, educational interventions are effective strategies to improve healthcare providers’ knowledge, skills, attitudes and teamwork. The usefulness of low-fidelity manikins is commendable due to their global, cost-effective utility. Public trust can be improved with a competent healthcare team. Rural healthcare providers’ medical emergency readiness may reduce the gap between providing urban and rural health care.

Moreover, the stakeholders can direct their outlook towards rural healthcare development to develop a safe community. Such intervention should make it mandatory for rural healthcare providers to maintain acquired readiness. It is also recommended that undergraduate and postgraduate curricula integrate emergency training programs to develop a strong workforce for the future. This review identified the global variations in rural healthcare providers’ emergency readiness. Most of the published studies are from higher-income countries. Despite the understanding that quality care is essential to reduce emergency-related ill health in low to middle-income countries, limited studies have focused on medical emergency readiness from those countries [16]. One of the reasons behind this situation is the lack of research priorities and interest in medical emergency readiness among low to middle-income countries. The identified research challenges are diagnostic uncertainties in medical emergencies, lack of interventions, lack of study design and data collection, lack of understanding of ethical considerations and inadequate research capacity [16]. However, evidence suggests that collaborative emergency care research support from high-income countries can strengthen emergency care research in low to middle-income countries [16]. Therefore, future research should focus on that direction.

However, there were several limitations associated with selected studies. The selected studies were limited to pre- and post-quantitative interventional studies, with full text published in the English Language, between 2013 and 2023 that followed PICO strategies within the selected databases. There could be studies about emergency medical readiness in other languages. Grey literature was also excluded from the review, although that could have contributed to rural healthcare providers’ emergency readiness. Two studies were RCT, and one study reported failure to maintain randomisation at the end of the study, while both studies did not address blinding the case and control in RCT. Other studies were quasi-experimental and did not explain the randomisation procedures. Furthermore, most studies did not report the behavioural outcomes, and most have not explained the psychometric properties of the outcome measures. Therefore, the methodological limitations associated with the selected studies remind the readers to interpret the result with a sensible conclusion.

Other limitations were the inclusion of heterogeneous participants from varying disciplines and attrition bias. The participants’ background knowledge, scope of practices and clinical expertise varied. So, the results could have been different if the training had been designed for a particular group of healthcare providers, such as nurses, doctors, or clinical assistants. Education is essential; however, if it is tailored to the unique needs of healthcare providers, it will be more effective in providing the best possible care for patients than conducting a general education [55].

## Conclusion

The review identified a global knowledge, skills, and competency gap in rural healthcare providers’ medical emergency readiness. At the same time, all interventions effectively enhanced rural healthcare providers’ medical emergency readiness. The acquired skills, knowledge, and competencies need to be maintained after the training. A regular in-service emergency training program should integrate with mandatory training for healthcare providers to achieve the above aims. There needs to be more research focused on educational intervention in rural healthcare providers’ medical emergency readiness despite trauma-related high mortality rates from low to middle-income countries; future research should be focused on the effectiveness of the educational interventions among those countries. Collaborative research development will be an effective strategy. Future research should focus on this direction.

Moreover, the selected studies did not evaluate the behavioural outcome in clinical settings. Therefore, future research should also be directed to understanding the clinical outcome of the interventions in rural settings. Most of the identified papers used a quasi-experimental design to assess the effectiveness of the intervention. A randomised controlled trial on medical emergency training can yield the most robust research findings. Therefore, we recommend randomised control trials with large samples to understand the outcome of medical emergency training and associated retention of the medical emergency readiness of rural healthcare providers. The review results identified that the interventions positively impacted medical emergency readiness globally. Furthermore, educational intervention with low-fidelity simulation was cost-effective in higher and low-middle-income countries. However, the results should be interpreted cautiously due to the limitations of the selected studies.

### Electronic supplementary material

Below is the link to the electronic supplementary material.

Supplementary Material 1

Supplementary Material 2

Supplementary Material 3

## Data Availability

No datasets were generated or analysed during the current study.
