# Supplementary material for: Efficacy of educational interventions on improving medical emergency readiness of rural healthcare providers: a scoping review
Source: BMC Health Serv Res. 2024 Jul 25;24:843. doi: 10.1186/s12913-024-11116-7 (PMC11282721; doi:10.1186/s12913-024-11116-7)
Supplement: Supplementary file 1 — Supplementary Material 1 [file 12913_2024_11116_MOESM1_ESM.docx]

**Appendix 1: 1 PICO STRATEGY**

| **Categories** | **Criteria** | **Search strategies** |
| --- | --- | --- |
| Population | Rural Health care providers | rural OR remote “health care providers “OR “health care clinicians” OR “doctors” OR “nurses” OR “Health care assistants” |
| Intervention | Quantitative experimental studies: Where health care providers have undergone training or education with pre and post-test assessment | "Educational intervention" OR "training" OR "basic life support training" OR "Evidence-based practices" |
| Comparison | Use outcome measures to assess the emergency medical readiness of healthcare providers. | Medical emergency readiness OR competence OR skill OR knowledge or confidence |
| Outcome | Identify the changes due to the intervention | Effect of intervention OR training OR education OR evidence-based practice OR basic life support*. |
