# Supplementary material for: Efficacy of educational interventions on improving medical emergency readiness of rural healthcare providers: a scoping review
Source: BMC Health Serv Res. 2024 Jul 25;24:843. doi: 10.1186/s12913-024-11116-7 (PMC11282721; doi:10.1186/s12913-024-11116-7)
Supplement: Supplementary file 2 — Supplementary Material 2 [file 12913_2024_11116_MOESM2_ESM.docx]

**Appendix 2: Critical appraisal of the selected Quasi experimental studies**

| Author & year | Q1 | Q2 | Q3 | Q4 | Q5 | Q6 | Q7 | Q8 | Q9 |
| --- | --- | --- | --- | --- | --- | --- | --- | --- | --- |
| Cullinane et al. (2022) | y | y | n | n | y | y | y | y | y |
| Kivlehan et al. (2021) | y | y | n | n | y | y | y | y | y |
| Menon et al. (2020) | y | y | n | n | y | y | y | y | y |
| Gutenstein, Kiuru and Withington (2019) | y | y | n | n | y | y | y | y | y |
| Monachino et al. (2019) | y | y | n | n | y | y | y | y | y |
| Ferguson et al. (2018) | y | y | n | n | y | y | y | y | y |
| Naidoo (2017) | y | y | n | n | y | y | y | y | y |
| Shenoi et al. (2013) | y | y | n | n | y | y | y | y | y |

Note: Yes = Y, No = N, Unclear = U, Not Applicable = NA.

Q1 = is it clear for the study what is the cause and what is the effect? Q2 = Were the participants included in any comparisons similar?; Q3 = Were the participants included in any comparisons receiving similar treatment, other than the exposure or intervention of interest?; Q4 = Was there a control group?; Q5 = Were there multiple measures of outcomes?; Q6 = Was follow complete?; Q7 = Were the outcomes of participants included in any comparisons measured in the same way?; Q8 = Were outcomes measured in reliable way?; Q9 = Was appropriate statistical analysis used?
