# Supplementary material for: Efficacy of educational interventions on improving medical emergency readiness of rural healthcare providers: a scoping review
Source: BMC Health Serv Res. 2024 Jul 25;24:843. doi: 10.1186/s12913-024-11116-7 (PMC11282721; doi:10.1186/s12913-024-11116-7)
Supplement: Supplementary file 3 — Supplementary Material 3 [file 12913_2024_11116_MOESM3_ESM.docx]

**Appendix 3: Critical appraisal of the selected Randomised Controlled Trial studies**

| Author & year | Q1 | Q2 | Q3 | Q4 | Q5 | Q6 | Q7 | Q8 | Q9 | Q10 | Q11 | Q12 | Q13 |
| --- | --- | --- | --- | --- | --- | --- | --- | --- | --- | --- | --- | --- | --- |
| Christiansen et al. (2023) | y | n | y | n | n | y | y | y | y | y | y | y | y |
| Stellflug and Lowe (2018) | y | u | y | y | y | y | y | y | y | y | y | y | y |

Note: Yes = Y; no = N; unclear = U; and not applicable = NA.

Q1 = Was true randomisation used for the assignment of participants to the treatment group? Q2 = Was allocation to treatment-to-treatment groups concealed? Q3 = Were treatment groups similar at the baseline? Q4 = Were participants blind to treatment assignment? Q5 = Were those delivering treatment blind to treatment assignment? Q6 = Were outcomes assessors blind to treatment assignment? Q7 = Were the treatment group treated identically other than the intervention of interest? Q8 = Was follow-up complete? Q9 = Were participants analysed in the group to which they were randomised? Q10 = Were outcomes measured in the same way for treatment groups? Q11 = Were outcomes measured in a reliable way? Q12 = Was appropriate statistical analysis used? Q13 = Was the trial design appropriate?
